# The use of human decellularized amniotic membrane as pulmonary valve leaflets in right ventricular outflow tract reconstruction – an *in vivo* proof of concept study

**DOI:** 10.3389/fbioe.2026.1735821

**Published:** 2026-02-18

**Authors:** Mohamed T. Ghorbel, Tasneem Salih, Giulia Parolari, Katie L. Skeffington, Sofia Di Leonardo, Danila Vella, Gaetano Burriesci, Massimo Caputo, Dominga Iacobazzi

**Affiliations:** 1 Translational Health Sciences, University of Bristol, Bristol, United Kingdom; 2 Department of Cardiac Surgery, Bristol Royal Hospital for Children, Bristol, United Kingdom; 3 Ri.MED Foundation, Palermo, Italy; 4 Deptartment of Engineering, University of Palermo, Palermo, Italy; 5 UCL Mechanical Engineering, University College London, London, United Kingdom

**Keywords:** amniotic membrane, pre-clinical study, pulmonary valve, right ventricular outflow tract reconstruction, swine model

## Abstract

**Introduction:**

Despite fundamental improvements in surgical treatment of Congenital Heart Defects, there are still challenges related to premature failure of the material used for such corrections, thus resulting in repeated operations during a patient’s life. This is particularly the case for complex defects with Right Ventricular Outflow Tract (RVOT) obstruction, such as in Tetralogy of Fallot/Pulmonary Atresia, whereby the pulmonary valve reconstruction remains problematic due to short-term durability of the currently used replacement solutions. We set out to test, for the first time, the suitability of amniotic membrane derived from human placenta for use in cardiovascular replacement of pulmonary valve.

**Methods:**

The decellularized and preserved amniotic membrane, obtained through our optimised protocol, was characterised for mechanical and hydrodynamic properties *in vitro*, and then implanted in the RVOT position of two Landrace piglets for *in vivo* feasibility and performance evaluation.

**Results:**

Both the *in vitro* and *in vivo* assessments showed favourable outcomes. The decellularized amniotic membrane had mechanical properties comparable to the native porcine pulmonary valve leaflets. In hydrodynamic testing, the decellularized amniotic membrane-made valve exhibited favourable opening dynamics, with smooth and coordinated leaflet motion throughout the cycle. *In vivo*, the decellularized amniotic membrane-based valved conduit showed patency in the short- and long-term with no sign of stenosis or regurgitation.

**Discussion:**

This study provides an *in vivo* proof of concept that the decellularized amniotic membrane can be implanted and perform as functional pulmonary valve in a porcine animal model mimicking the clinical scenario of Tetralogy of Fallot surgical correction in infants.

## Introduction

Congenital heart disease (CHD) is the most common congenital malformation in humans worldwide, accounting for 1.35 million births annually (Jacobs et al., 2014; Drury et al., 2022; Xu et al., 2025). Severe CHDs, mainly involving the right ventricular outflow tract (RVOT), including the pulmonary artery (PA) and valve (PV), are commonly repaired through surgery within the first year of life. Owing to the advancements in surgical techniques and medical technologies, infant mortality from CHD has declined rapidly within the last 30 years (GBD 2017 Congenital Heart Disease Collaborators, 2020; Bouma and Mulder, 2017). However, in complex defects with RVOT obstruction, such as in Tetralogy of Fallot/Pulmonary Atresia, the PV preservation is frequently impossible, and its reconstruction remains problematic in this paediatric patient population.

Over the past 7 decades, many materials have been used for the replacement of the PV, including valved bovine jugular vein, decellularized pulmonary allografts, non-stented xenografts or stented porcine valves. However, none of these materials has proven lifelong durability, with the main limitations being fast degeneration and lack of growth potential, in addition to fibrosis and calcification (Herrmann and Brown, 2020; Almeida-Pinto et al., 2023). Young patients are therefore committed to multiple reoperations for successive larger valve replacements until they reach adulthood. Nonetheless, repeated valve replacement in children and young patients has been shown to have major implications for their quantity and quality of life, which are both negatively affected (Kvidal et al., 2000; Sharabiani et al., 2016).

Opportunities for more viable valve alternative lie in the development of innovative valve designs and materials that would offer cardiovascular surgeons a definitive solution for PV surgical reconstruction, with long-lasting benefit for both the young patients’ quality of life and the finances of the national healthcare system.

To address this problem, our group has investigated the potential of the human placenta-derived Amniotic Membrane (AM) to be used as a durable biocompatible valve leaflet in the RVOT reconstruction, overcoming the limitations of the currently used valve replacement prosthesis.

The clinical use of the AM dates back to the beginning of the 20th century when it was first used as a superficial skin dressing (Lim R., 2017), however it was not until the last 2 decades that it has gained increased popularity in a wide range of tissue engineering applications, owing to its excellent biological properties that enhance both tissue healing and regeneration (Munoz-Torres et al., 2022).

Primarily employed as scaffolding material in the wound healing of soft tissues like skin and cornea (Jirsova and Jones, 2017), the AM has then broadly been applied in many other clinical procedures, including gynaecology (Lau et al., 2020), gastrointestinal surgery (Ratto et al., 2022), orthopaedics (Demirkan et al., 2022), and wound dressing for burns or the acute and chronic wounds (Song et al., 2017).

Moreover, AM continues to gain attraction as a novel material in the cardiac tissue engineering field, especially as a scaffold for blood vessels (Lee et al., 2012; Swim et al., 2019) and pericardial substitute (Marsh, 2017; Francisco et al., 2016), as well as myocardial infarction treatment in the form of patches or injectable AM-derived gel matrix (Jiang et al., 2021; Lim JJ., 2017).

To the best of our knowledge, our work is the first to employ the AM-derived scaffold as biocompatible, durable leaflet for the PV reconstruction in a large animal model mimicking conditions whereby a definitive reparative solution does not yet exist, such as Tetralogy of Fallot/Pulmonary Atresia. In the current work, we have provided a proof of concept that, once implanted in the PV position of our porcine model of RVOT reconstruction, the AM, decellularized and preserved by our unique method, performs in a physiological manner, with normal blood velocity, in both short- and long-term assessments. In addition, we have shown favourable outcome when our AM-derived scaffold was tested *in vitro* for mechanical and hydrodynamic functions.

## Methods

### Ethics

Human placenta was collected from patients in compliance with the Human Tissue Act, with patients’ consent under NHS ethics license (REC ref. 23/WA/0119).

### Amniotic membrane tissue preparation, decellularization and preservation

AM was collected from human caesarean sections. Two different human placentas were used in this study; one for each experimental animal. AM was removed from the fetal side of the placenta by blunt dissection and washed repeatedly in PBS/1% Penicillin-Streptomycin (PS). It was then stored overnight at 4 °C in PBS/1% PS until decellularization was performed as previously described (Swim et al., 2019).

Briefly, the AM tissue was placed in 0.1% Trypsin in PBS/1% PS solution and incubated for 3 h at 37 °C under gentle rotation. The solution was changed to 500 U/mL DNase in PBS/1% PS buffer and incubated for an additional 3 h at 37 °C under gentle rotation. Decellularized AM (d-AM) was then washed in PBS/1% PS every day for 5–9 days at 37 °C under gentle rotation.

AM and d-AM were preserved by freezing at −20 °C, then freeze-drying and storing at room temperature.

### Hydrodynamic testing

**FIGURE 1:**
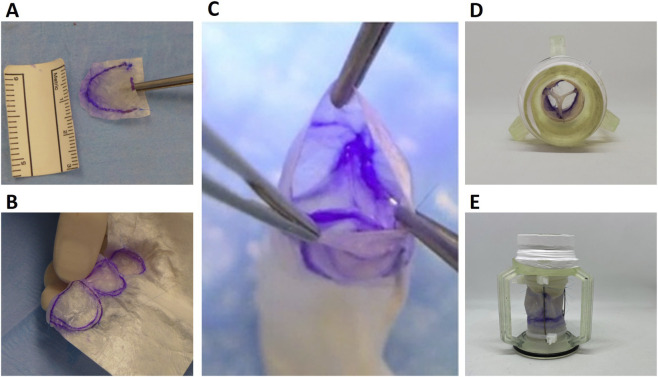
d-AM-based valve leaflets and valved conduit design. **(A)** d-AM shaped into one leaflet. **(B)** d-AM tri-leaflets stitched to ProxiCor® conduit. **(C)** Macroscopic view of the final valved conduit. Photographs of the top **(D)** and lateral **(E)** views of the valve prior to being assembled in the pulse duplicator for hydrodynamic testing.

A d-AM-based valved conduit with an internal diameter of 18 mm and a length of 35 mm was prototyped (Figures 1A–C). The valve leaflets were constructed from amniotic tissue and integrated into a conduit support made of porcine-derived small intestinal submucosa (SIS) scaffold (ProxiCor®). The valved conduit was mounted on a custom-made 3D-printed resin holder (Figures 1D,E), designed to minimize distortion during handling and to facilitate positioning within the hydro-mechanical pulse duplicator system (ViVitro Superpump, SP3891, Canada).

The ViVitro system is an *in vitro* cardiovascular simulator capable of reproducing physiologic pressure and flow conditions of the heart chambers and great arteries. It consists of a servo-controlled pump, compliance chambers, and peripheral resistance elements, enabling accurate replication of pulsatile flow and pressure waveforms. In the setup, the AM-based valved conduit was positioned in the arterial outflow section, while an Abbott Masters Series bi-leaflet mechanical valve (size 29) was placed in the atrioventricular section.

Operating conditions were set according to international standard ISO 5840 guidelines to represent right-heart function in toddlers. Specifically, simulations were run at a heart rate of 100 bpm, a mean cardiac output of 3 L/min, a pulmonary mean pressure of 20 mmHg, and a systolic duration (defined as the phase of ventricular volume decrease) corresponding to 45% of the cardiac cycle.

Phosphate-buffered saline at room temperature was used as the test fluid. Pressures in the atrial, ventricular, and pulmonary chambers were measured with Mikro-Cath pressure catheters (Millar, Houston, TX, United States), while flow through the d-AM valved conduit was monitored using an electromagnetic flow probe (Carolina Medical Electronics, East Bend, NC, United States) placed at its inflow. Pressure and flow signals were recorded over ten consecutive cycles, from which the mean transvalvular pressure difference and the effective orifice area were evaluated and averaged, as recommended in the ISO 5840 standard.

Specifically, the mean transvalvular pressure difference, Δp , was calculated during the positive differential pressure period. The effective orifice area, expressed in cm^2^, was derived from flow and pressure data, based on the revised Gorlin’s formula recommended in the ISO 5840:

$$A_{EO}=\frac{q_{V_{RMS}}}{51.6\;\sqrt{\frac{\Delta p}{\rho }}}$$

with $q_{V_{RMS}}$ corresponding to the root mean square forward flow measured during the positive differential pressure period, and ρ to the density of the test fluid, expressed in g/cm^3^.

### Mechanical testing

Samples were analysed for mechanical properties with pneumatic grips and a 100 N load cell on an Instron 3343B machine. Crosshead speed was 10 mm/min. Samples were measured for tensile stress at break and Young’s Modulus using Bluehill software (Instron). Porcine PV leaflets were used as control. These were obtained from piglets sacrificed for other ethically approved studies, to ensure that no animals were used for this sole purpose.

### Histology and immunohistochemistry

To assess the occurred decellularization of the AM, and then the d-AM leaflet repopulation by endogenous cells after implantation, tissues were fixed in 4% paraformaldehyde (PFA) and embedded in paraffin in a Thermo HistoStar machine. Sections were cut on a microtome (Thermo) at 5 mm, floated onto Menzel-Glaser Super Frost Plus slides (Thermo), and dried overnight.

For histological analysis, hematoxylin and eosin (H&E) staining was performed using a Shandon Varistain 24–4 (Thermo Fisher Scientific) automated machine. For immunofluorescence analysis, samples were deparaffinized in Clearene and rehydrated through an alcohol gradient. Antigen retrieval was performed with 10 mM citrate buffer pH 6.0 heated to boil. 10% goat serum (Sigma-Aldrich) in PBS was used to block the samples for 30 min at room temperature. Samples were incubated with the primary antibody (Vimentin 1:100, Abcam) overnight at 4 °C. Secondary antibody [1:400 goat-anti-rabbit-Alexa Flour 546 (Abcam)] was then incubated on the sections for 1 h at room temperature in the dark. DAPI was used to counterstain the nuclei, and the slides were mounted using HardSet mounting medium (VECTASHIELD). Images were taken with a Zeiss Observer.Z1 fluorescent microscope. ImageJ software was used to quantify the vimentin expression in the tissue sections.

### *In vivo* proof of concept

Three 3-week-old female Landrace pigs of 10–15 kg were used in this study. One Sham animal went through cardiopulmonary bypass but without RVOT/PV replacement. Two experimental animals went through cardiopulmonary bypass with RVOT/PV replacement. No mortality was recorded. Animals were treated in accordance with the ‘‘Guide for the Care and Use of Laboratory Animals’’ published by the National Institutes of Health in 1996 and conforming to the United Kingdom’s ‘‘Animals Act’’ published in 1986, under the United Kingdom Home Office project licence PP0950206.

Surgical procedures were performed under general anaesthesia and neuromuscular blockade (Pancuronium Bromide; 2 mg/mL). Anaesthesia was induced by intramuscular injection of 15 mg/kg Ketamine, 0.4 mg/kg Midazolam, and 5 mg/kg Dexmedetomidine, and then maintained with inhalation of 1%–2% Isoflurane. Right before the surgery, 2D standard transthoracic echocardiography (TTE) and Doppler echocardiography were performed followed by the creation (for experimental group) of single layered d-AM tri-leaflets stitched to the ProxiCor® scaffold, forming a conduit with 16 mm diameter and 70 mm length. The heart was accessed by median sternotomy followed by connecting cardiopulmonary bypass to the heart by cannulating the ascending aorta, superior vena cava (directly) and inferior vena cava via the right atrial appendage. For experimental group and whilst the heart was beating, native RVOT/PV were excised and replaced by the engineered valved conduit. After the surgery, the piglet was extubated and was kept in intensive care for 24 h. Opioids and NSAIDS were administered to the piglet to manage pain whilst blood pressure, heart rate, oxygen saturation, temperature and chest drain output were checked every 2–4 h. The Sham pig was followed up for 6 months and assessed by 2D standard TTE Doppler echocardiography. The experimental pigs were followed up for 4 weeks and 6 months 2D standard TTE and Doppler echocardiography was performed on the piglets to measure peak velocities and patency across the RVOT before explantation of the engineered valved conduit. At the indicated endpoints, the piglets were euthanized with an intravenous injection of 150 mg/kg of Pentobarbital sodium, followed by dissection of the heart to remove the tissue engineered valved conduit. The explanted valved conduit was then frozen in liquid nitrogen or fixed in 4% PFA before further immunohistochemical assessment.

### Statistical analysis

Statistical testing was carried out with t-test or one-way ANOVA with Tukey *post hoc* testing as appropriate. A value of p < 0.05 was considered statistically significant.

## Results

### d-amniotic membrane-based valve leaflets and valved conduit preparation and *in vitro* testing

Before *in vitro* and *in vivo* evaluation, the d-Amniotic Membrane was histologically assessed to confirm the removal of nuclei, as previously shown (Swim et al., 2019) (Supplementary Figure S1).

A porcine derived Small Intestinal Submucosa (SIS) scaffold (ProxiCor®) was made into a conduit, to which sheets of d-AM, shaped as three leaflets, were sewed to create the three leaflets (tricuspid) valved conduit (Figure 1). The resulting valved conduit was first tested in a pulse duplicator system. The prosthetic valve had a transvalvular pressure difference Δp of 8.836 ± 0.21 mmHg, and an effective orifice area of 1.03 ± 0.02 cm^2^. Although the ISO standard 5840 does not specify requirements for paediatric pulmonary devices, the estimated effective orifice area largely exceeds the threshold prescribed for larger-sized prosthetic aortic valves (a minimum $A_{EO}$ of 0.85 cm^2^ is requested for 19 mm diameter aortic valves). Moreover, the valve exhibited favourable opening dynamics, with smooth and coordinated leaflet motion throughout the cycle (Supplementary Video S1).

**FIGURE 2:**
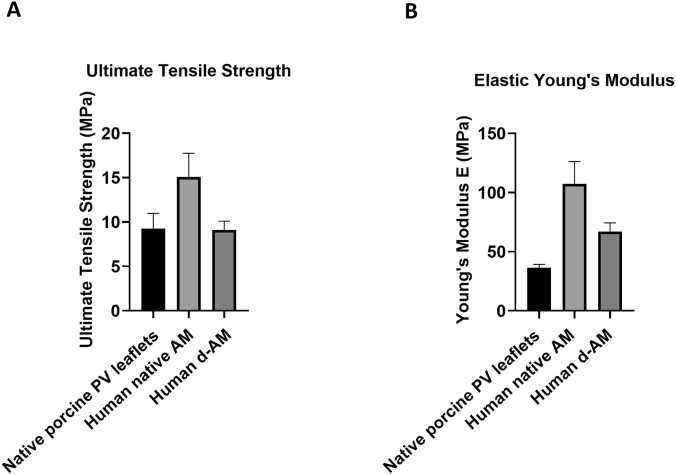
*In vitro* mechanical testing on the AM leaflet. Comparison in **(A)** Ultimate Tensile Strength and **(B)** Elastic Young’s Modulus between human native AM, human d-AM and native porcine PV leaflets. One-Way ANOVA followed by Tukey’s *post hoc* test (n = 3).

Mechanical testing was also conducted, to assess the strength and elasticity of the decellularized AM in comparison with the non-decellularised counterpart and the native porcine PV. The ultimate tensile strength (UTS) of the d-AM was comparable with the native porcine PV leaflets and lower than the native AM. Likewise, there was no significant difference in the Young’s modulus between the d-AM and the porcine PV, with both groups showing a lower value compared to the native AM (Figure 2).

### *In vivo* preclinical testing

We next assessed the feasibility and safety of using our d-AM based valved conduit in a unique model of porcine RVOT reconstruction developed by our group (Supplementary Figure S2).

One Sham piglet went through cardiopulmonary bypass surgery without RVOT/PV replacement and was followed up for 6 months. The PA was patent and the blood flow velocity through the PA was normal (V < 2 m/s) at 6 months post-procedure. No stenosis or regurgitation were detected (Supplementary Figure S3).

**FIGURE 3:**
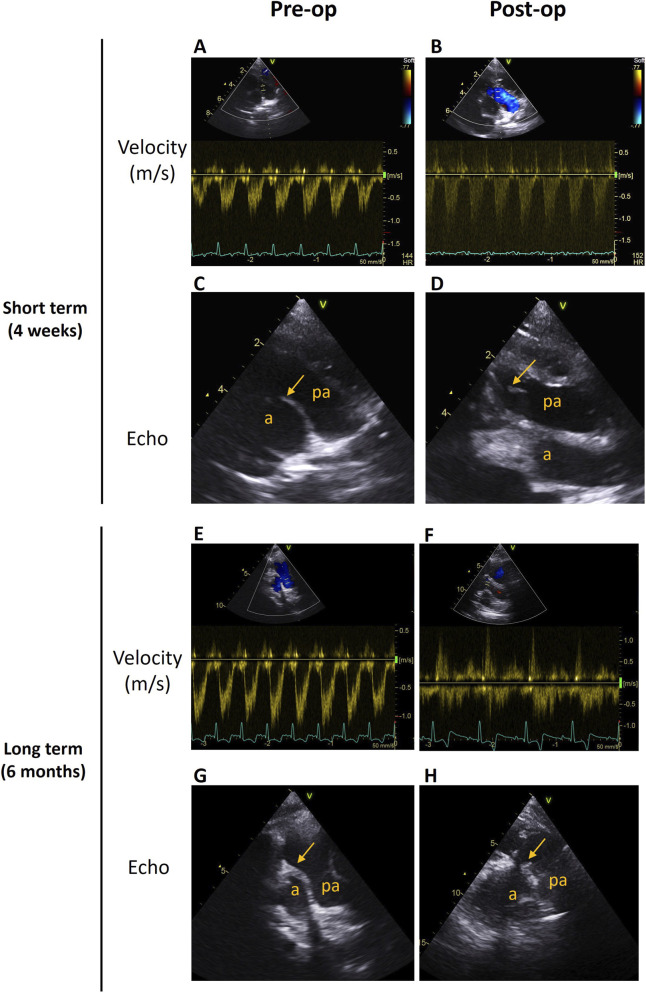
*In vivo* assessments of the d-AM leaflets animals at 4 weeks and 6 months post-operation. **(A,B)** Blood velocities through the PV/PA before surgery (pre-operation) and after 4 weeks. **(C,D)** Representative echocardiography (echo) images of the PA before and after (4 weeks’ time point) surgery (a, aorta; pa, pulmonary artery). **(E,F)** Blood velocities through the new valved conduit pre-operation and 6 months after surgery. **(G,H)** Representative echocardiography images of the PA before the operation and 6 months after, showing the d-AM leaflets indicated by an arrow (a, aorta; pa, pulmonary artery).

**FIGURE 4:**
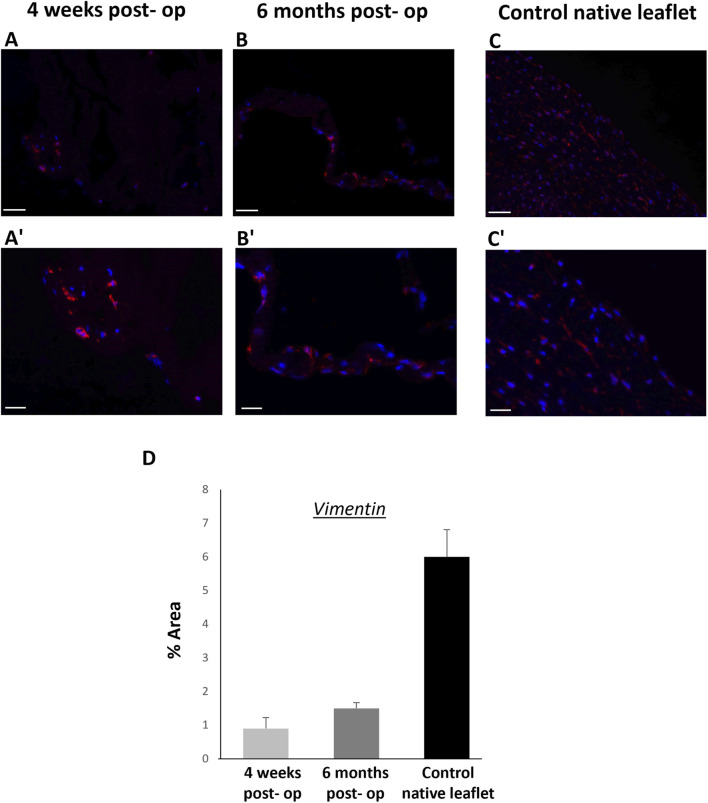
Immunohistochemistry of explanted d-AM-based and native PV leaflets. Representative images showing cross sections of the explanted d-AM leaflets, after 4 weeks and 6 months **(A, A’,B, B’)**. Newly formed layers of valvular interstitial cells (vimentin positive, red) repopulated the leaflets. Native PV leaflets were used as positive control **(C, C’)**. Scale bars = 20 μm (a, b, c), 10 μm (a’, b’, c’, higher magnification). **(D)** Bar chart showing the vimentin expression in the explanted d-AM leaflets, after 4 weeks, 6 months, and in the native PV leaflets. Each data point represents the average of 3 random fields of view per well.

Two piglets were implanted with d-AM valved SIS-conduits and followed up for 4 weeks and 6 months, for short- and long-term assessments, respectively. Similar to the Sham piglet, the PA of each experimental animal was patent and the blood flow velocity through the valved-conduit was normal (V < 2 m/s) at both time endpoints. No stenosis or regurgitation were detected (Figure 3). Additionally, immunohistochemical post-mortem assessment of the leaflets showed repopulation of the d-AM leaflets by endogenous valvular interstitial cells, supporting the remodelling potential and biocompatibility of the scaffold (Figure 4).

## Discussion

In the present study, we presented the human placenta amniotic membrane as suitable source of biocompatible scaffold for engineering cardiovascular replacement valves. Furthermore, we provided for the first time an *in vivo* proof of concept that the placenta-derived d-AM can be employed and can serve as a functionally active valve when implanted in the PV position of a porcine animal model mimicking the clinical scenario of Tetralogy of Fallot surgical correction in infants.

This study is a significant stepping stone towards achieving the goal of developing a growing valved conduit used as right ventricle/PA conduit in the repair of complex conditions in paediatric patients suffering from Tetralogy of Fallot with pulmonary stenosis or pulmonary atresia and truncus arteriosus.

In our previous work, we focused on the vascular component of such a graft, demonstrating, through a full scale randomised controlled pre-clinical trial, the safety, efficacy, and growing capacity of our mesenchymal stem cell (MSC)–seeded Small Intestinal Submucosa (SIS) tissue-engineered graft for main PA reconstruction in healthy piglets (Rapetto et al., 2022; Iacobazzi et al., 2024).

Due to the significant difference between the biological and biomechanical features of the vascular wall and the valvular leaflets, the same biomaterial (SIS) is unsuitable for the two parts of the graft. Indeed, when used for valve replacement in the aortic and mitral position, the SIS (ProxiCor [previously known as CorMatrix] Inc) showed little evidence of remodelling and no appearance of tissue resembling the native valve, in addition to high recurrence of severe mitral regurgitation (Zaidi et al., 2014; Kelley et al., 2017; Weis et al., 2022; Mosala et al., 2017). Other studies reported severe/moderate regurgitation or severe/mild-to-moderate insufficiency within the first year of implantation as pulmonary mono- or tri-cusp valve (Mosala et al., 2017; Witt et al., 2013; Scholl et al., 2010).

Several other materials have been used as valve substitutes, including autologous pericardium (with or without fixation), preserved xeno-pericardium, and various prosthetic materials (i.e., polyethylene terepthalate [Dacron] and polytetrafluoroethylene [Gore-Tex]). None of those biomaterials showed satisfactory results due to the presence of inflammatory reactions, aneurysm formation and calcification (Almeida-Pinto et al., 2023; Hickey et al., 2009; Breymann et al., 2009; Rajani et al., 1998). The most promising biomaterial for valve leaflet extension, so far, has been the glutaraldehyde-fixed autologous pericardium, which showed greater resistance to retraction and degeneration than the bovine counterpart; however, its poor growth potential still remains a major hurdle (Mihos et al., 2020; Duran et al., 1995; Kostyunin et al., 2020).

Therefore, the lack of solutions that would guarantee more viable and long-lasting valve alternative is still a concerning problem and the need for new technologies in PA and valve reparative operations in children with Tetralogy of Fallot spectrum has now been widely acknowledged by national and international health bodies.

With the ideal biomaterial being compatible with growth, pliable, resistant to tearing, calcification, and able to heal without scar formation, we have identified in the AM an optimal candidate for valve replacement, owing to all these peculiar characteristics (Leal-Marin et al., 2021; Munoz-Torres et al., 2022). Additionally, the biological proprieties of AM include those of immunomodulatory and anti-microbial, allowing it to elicit the desired function without generating any local or systemic adverse response in the native tissue (Munoz-Torres et al., 2022; Tehrani et al., 2017).

Yet, as the threat of immunogenicity, although low, still exists, we have optimized a unique decellularization protocol to minimize the risk of rejection, followed by a freeze/drying preservation method to create an off-the-shelf product suitable for clinical use (Swim et al., 2019). Noticeably, both the removal of cells and the preservation protocol did not alter the structural integrity and mechanical properties of the AM leaflets. This is an important achievement, considering that the failure after preservation of the “fresh” valves, mostly maintained at 4 °C in preservative solutions or cryopreserved, is one of the major hurdles of bioprosthetic heart valves (Peters et al., 2024).

Our decellularized preserved AM proved to provide adequate hydrodynamics when tested in the right heart setting of a pulse duplicator as per the international standard ISO5840, which is an essential prerogative for the regulatory requirements of valves and valved products to be used in clinical settings. Although the *in vitro* hemodynamic behaviour of a valve might differ from the one of an *in vivo* setting, due to the use of simplified fluids (saline instead of blood), fixed parameters (heart rate, cardiac output) and lack of physiological factors (calcification, immune response), the pulse duplicator systems are essential in the first stage of heart valve development and investigations to assess short term functionality, thus reducing the number of proof-of-concept animals, and to optimise the valve design under controlled, yet clinically relevant conditions.

To further validate the preliminary *in vitro* hydrodynamic findings, we have provided an *in vivo* proof of concept showing that the d-AM, sewn as a three leaflets valve within a porcine derived extracellular matrix SIS scaffold (ProxiCor®) -made conduit, retains its patency without showing any sign of stenosis or regurgitation, at both short and long-term assessments. Additionally, a noticeable sign of cell repopulation was detected on the explanted d-AM leaflets, although this did not result into a complete recellularization at the 6-month follow up. This is expected, considering the rapid growth rate of pigs, particularly during the grow-finish phase, which is significantly faster compared to the time required for valve recellularization, a process that might take 12 months, or more, to complete (Stoian et al., 2024; Vafaee et al., 2022). A longer-term longitudinal investigation, currently underway, as a follow-up to the current proof of concept study, would help us understand more about the resultant tissue recellularization and provide further insight on the cellular composition and structure of the cell-repopulated d-AM leaflets.

## Limitations

Although the proof of concept of feasibility for implantation of our decellularized amniotic membrane as pulmonary valve replacement in paediatric Tetralogy of Fallot correction showed favourable outcome, the small sample size constitutes a limitation of our work.

A sample size of two animals does not allow to draw any definitive conclusion on the benefit of our strategy, however, the exploratory nature of this study enabled the validation of our porcine model for pulmonary valve reconstruction and the refinement of the surgical procedure, in alignment with the 3Rs (Replacement, Reduction, Refinement) principle, before committing to a larger study. This is currently underway, and has been designed as a full scale randomised and statistically powered pre-clinical study, with which we are aiming to provide a proof of safety and efficacy of our novel d-AM-based leaflets for PV reconstruction.

This is a fundamental requirement for the clinical translation of our proposed solution into the first-in-human clinical trial. Our ultimate aim is to combine the previously developed MSCs–seeded SIS conduit with the d-AM leaflets to create a tissue engineered valved conduit, which would offer the paediatric cardiac tissue engineering community a long-lasting alternative to complex CHD corrections, such as Tetralogy of Fallot/Pulmonary Atresia where full RVOT reconstruction (including PA and PV) is required. Currently, there is no definitive solution to correct those CHDs therefore, such an alternative is much needed.

## Perspectives

Once the safety and efficacy of our combined proposed technology will be proved, this will be translated into the equivalent clinical grade, GMP-compliant product. Parallelly, ethical approval by the national regulatory body for the first-in-human clinical trial will be sought, by submitting all the relevant documents, such as the Protocol, Investigator’s Brochure (IB), Informed Consent Document (ICD) (Supplementary Figure S4).

We envision this approach, of using decellularized amniotic membrane-made leaflets in pulmonary valve reconstruction, to be of great interest and relevance in tissue engineering field applied to paediatric valvular defects correction, as it may open up new avenues for definitive treatment of such congenital cardiac defects, with remarkable medical and social benefits.

## Data Availability

The raw data supporting the conclusions of this article will be made available by the authors, without undue reservation.

## Supplementary material

SUPPLEMENTARY FIGURE S1 Histological assessment of the AM leaflet. Representative images showing the nuclei content in the human native AM **(A, C)** and human d-AM **(B, D)** via H&E **(A, B)** and DAPI **(C, D)** staining. Scale bars = 20 μm.

SUPPLEMENTARY FIGURE S2 Macroscopic images of the d-AM valved conduit. Views **(A)** during implantation and **(B)** after explantation. Arrows pointing the leaflets.

SUPPLEMENTARY FIGURE S3
**(A, B)** Blood velocities through PV of the Sham pig pre-operation and at 6 months of follow-up. **(C, D)** Representative echocardiography images of the Sham pig’s PA before the operation and 6 months after (a, aorta; pa, pulmonary artery).

SUPPLEMENTARY FIGURE S4 Schematic diagram showing the experimental design. During the Research and Development stage, an effective decellularization and preservation protocol to process the amniotic membrane (AM) has been established and optimised. The resulting decellularised AM (d-AM) valve leaflets have been histologically characterised and *in vitro* tested to assess the mechanical and hydrodynamic properties. The following Pre-clinical study has been designed as an initial Exploratory Phase, to provide a Proof of Concept of Feasibility for implantation of the d-AM leaflets in a porcine model of pulmonary valve reconstruction, and a subsequent Confirmatory Phase, statistically powered, for Proof of Safety and Efficacy. The completion and success of the Pre-clinical study will lead into its clinical translation stage, which will consist in the clinical grade manufacturing of the d-AM, following the GMP compliant standards, and in the preparation and submission of all the documentation required for trial approval by the regulatory bodies.
