# Supplementary figures and images for: The use of human decellularized amniotic membrane as pulmonary valve leaflets in right ventricular outflow tract reconstruction – an in vivo proof of concept study
Source: Front Bioeng Biotechnol. 2026 Feb 18;14:1735821. doi: 10.3389/fbioe.2026.1735821 (PMC12957159; doi:10.3389/fbioe.2026.1735821)

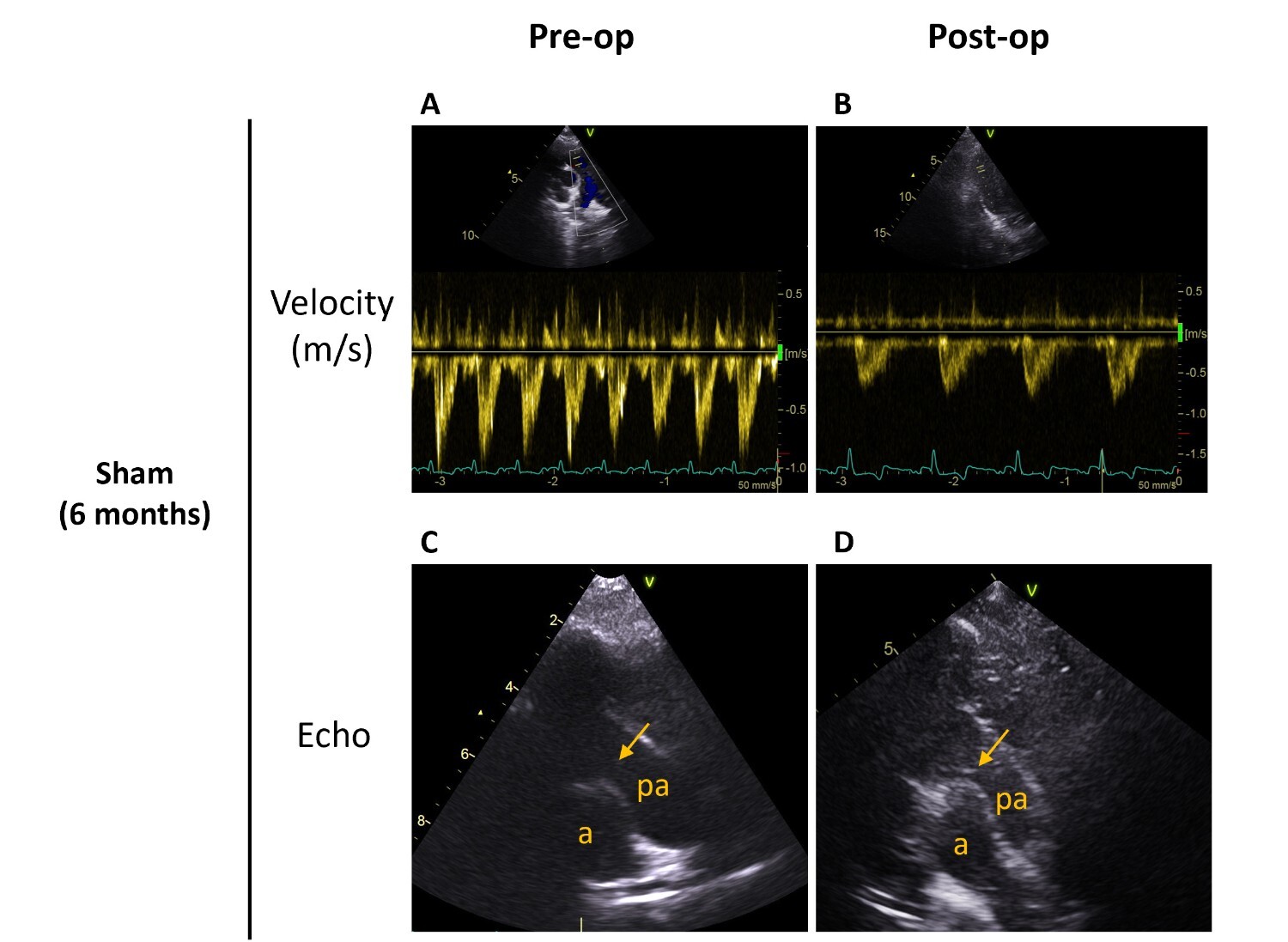

Supplement: Supplementary file 1 [file Image3.jpeg]

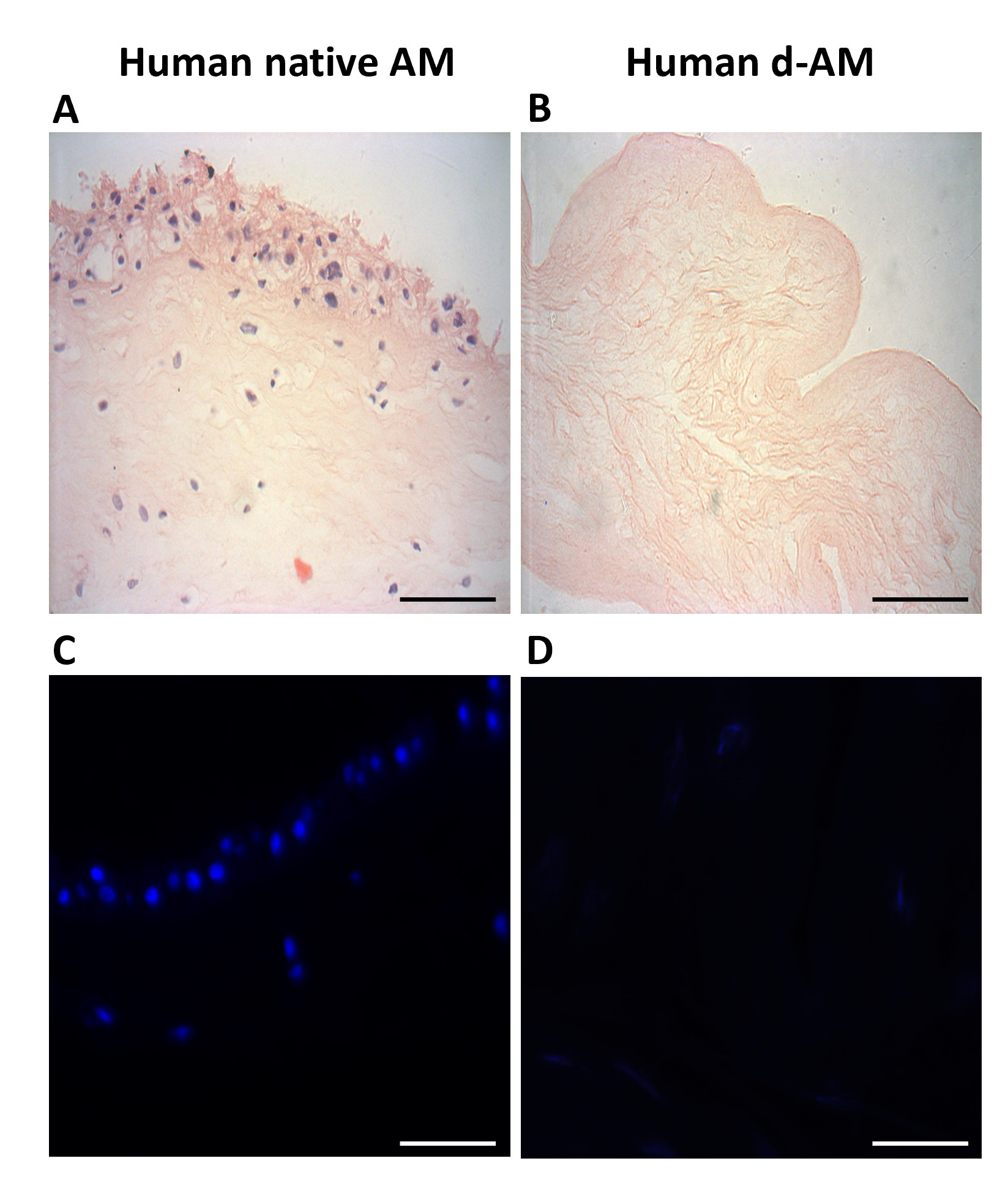

Supplement: Supplementary file 2 [file Image1.jpeg]

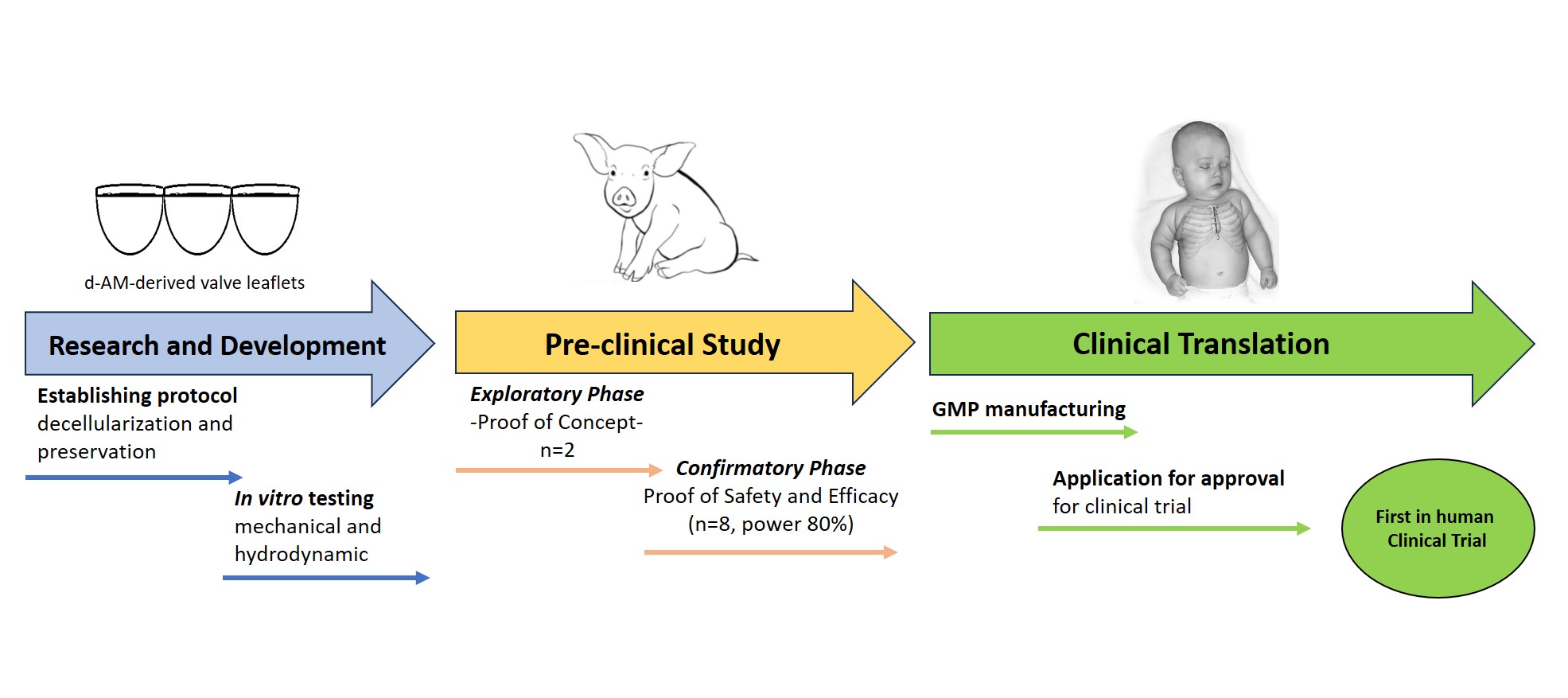

Supplement: Supplementary file 3 [file Image4.jpeg]

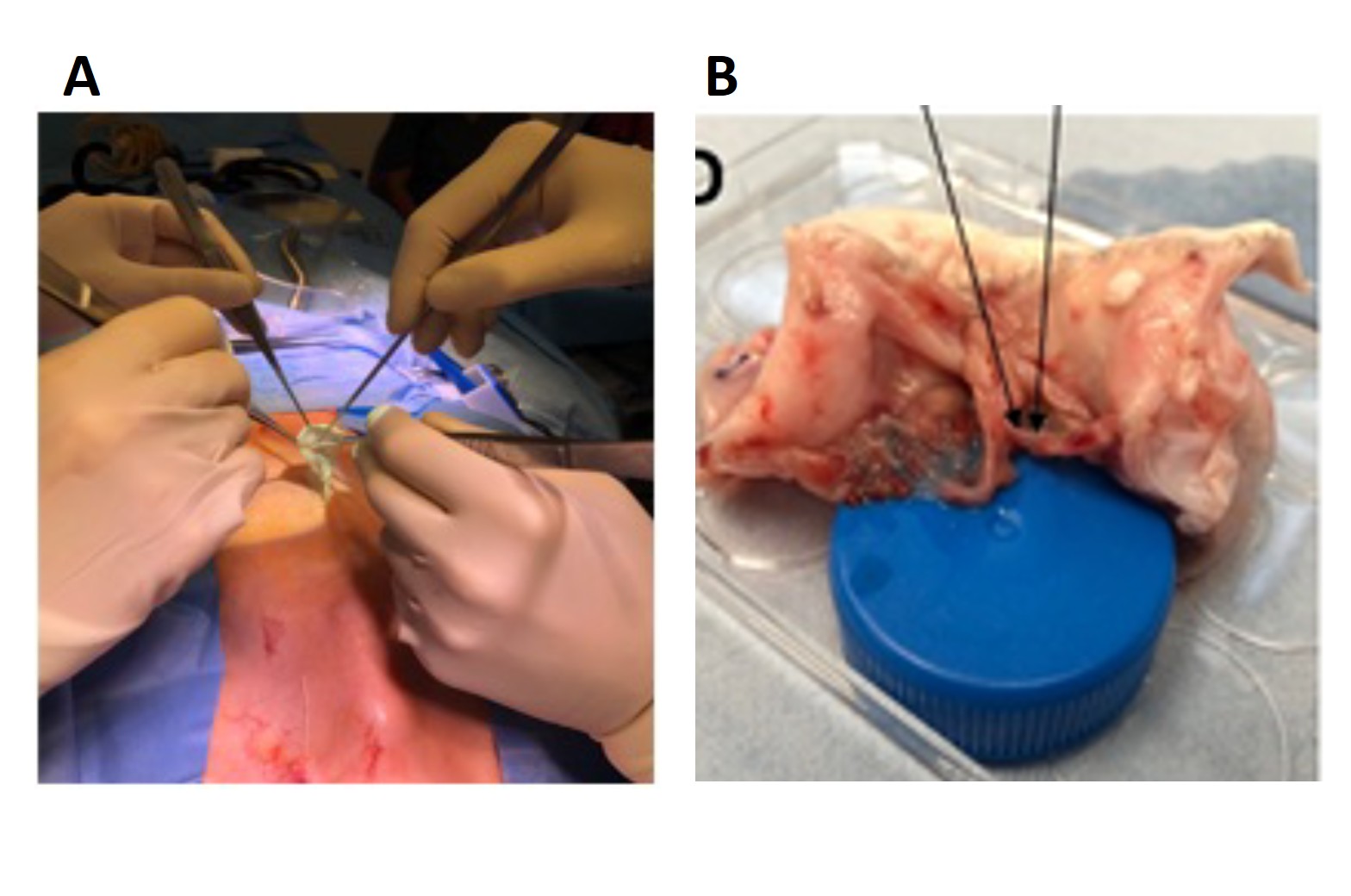

Supplement: Supplementary file 4 [file Image2.jpeg]
